# Low‐dose decitabine promotes M2 macrophage polarization in patients with primary immune thrombocytopenia via enhancing KLF4 binding to PPARγ promoter

**DOI:** 10.1002/ctm2.1344

**Published:** 2023-07-24

**Authors:** Xia Shao, Pengcheng Xu, Lili Ji, Boting Wu, Yanxia Zhan, Xibing Zhuang, Yang Ou, Fanli Hua, Lihua Sun, Feng Li, Xiangdong Wang, Hao Chen, Yunfeng Cheng

**Affiliations:** ^1^ Center for Tumor Diagnosis and Therapy Jinshan Hospital Fudan University Shanghai China; ^2^ Department of Hematology Zhongshan Hospital Qingpu Branch Fudan University Shanghai China; ^3^ Department of Transfusion Medicine Zhongshan Hospital Qingpu Branch Fudan University Shanghai China; ^4^ Institute of Clinical Science Zhongshan Hospital Fudan University Shanghai China; ^5^ Department of Thoracic Surgery Zhongshan‐Xuhui Hospital Fudan University Shanghai China

**Keywords:** decitabine, krüppel‐like factor 4, m2 macrophages, peroxisome proliferator‐activated receptor‐γ, primary immune thrombocytopenia

## Abstract

**Background:**

The first‐line therapy is effective for the treatment of primary immune thrombocytopenia (ITP); however, maintaining the long‐term responses remains challenging. Low‐dose decitabine (DAC) has been adopted to treat refractory ITP, while its role in macrophage polarization has not been fully understood. We aimed to investigate the mechanistic role of DAC in M2 macrophage polarization and evaluated its therapeutic effect in ITP.

**Methods:**

The M2 monocytes were identified by flow cytometry from peripheral blood mononuclear cells in healthy controls (HCs) and ITP patients. The expression of PPARγ, Arg‐1, DNMT3b and NLRP3, together with IL‐10 plasma levels was measured to examine its function. Bisulfite‐sequencing PCR was used to evaluate the methylation status of PPARγ promoter, and the binding affinity of KLF4 was measured by Cut&Tag. A sh‐PPARγ THP‐1 cell line was created to verify if low‐dose DAC‐modulated M2 macrophage polarization was PPARγ‐dependent. The passive ITP models were used to investigate the therapeutic effects of low‐dose DAC and its role in modulating polarization and immunomodulatory function of macrophages. NLRP3 inflammasome and reactive oxygen species were also tested to understand the downstream of PPARγ.

**Results:**

The M2 monocytes with impaired immunoregulation were observed in ITP. After high‐dose dexamethasone (HD‐DXM) treatment, M2 monocytes increased significantly with the elevated expression of PPARγ, Arg‐1 and IL‐10 in CR patients. Low‐dose DAC promoted M2 macrophage polarization in a PPARγ‐dependent way via demethylating the promoter of PPARγ, especially the KLF4 binding sites. Low‐dose DAC alleviated ITP mice by restoring the M1/M2 balance and fine‐tuning immunomodulatory function of macrophages. The downstream of the PPARγ modulation of M2 macrophage polarization might physiologically antagonize NLRP3 inflammasome.

**Conclusions:**

Low‐dose DAC promoted M2 macrophage polarization due to the demethylation within the promoter of PPARγ, thus enhanced the KLF4 binding affinity in ITP.

## Graphical abstract

Low‐dose DAC promoted M2 polarization via KLF4‐PPARγ‐NLRP3 pathway: Via demethylating the promoter of PPARγ, low‐dose DAC enhanced its binding with KLF4. By suppressing NLRP3 via the enhanced expression of PPARγ, DAC could promote M2 polarization more readily. In addition, alleviating ROS production might be another mechanism of DAC.

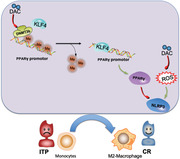

## 1 INTRODUCTION

Primary immune thrombocytopenia (ITP), as an autoimmune hemorrhagic disease, is characterized by excessive platelet devastation and impaired platelet production.^1^, ^2^ According to 2019 American Society of Hematology guidelines for ITP,^3^ the initial therapy for adult ITP includes daily prednisone or four‐day dexamethasone regimen.^4^ In patients who are corticosteroids‐dependent or have no response to corticosteroids, thrombopoietin receptor agonists are recommended as a second‐line regimen. However, long‐term remission in adult ITP is still challenging due to the risk of relapse and intolerance to prolonged medication. Recent evidence has revealed that decitabine (DAC), a demethylation regimen for myelodysplastic syndrome,^5^ could increase platelet count in ITP. Low‐dose DAC has been adopted to treat refractory ITP and demonstrated a satisfying prospect in clinical trails.^6^, ^7^ Although the impacts of DAC on T cells, myeloid‐derived suppressor cells (MDSC) and megakaryocytes have been studied,^8^, ^9^ the underlying mechanism of its effect on macrophages remains obscure.

The polarization of monocytes/macrophages, which is capable of remarkable plasticity, has been demonstrated to assume a significant part in various autoimmune diseases.^10^ Classically activated macrophages (M1) and alternatively activated macrophages (M2) are the two main states of tissue macrophages.^11^, ^12^ The tendency towards M1 polarization has been revealed to contribute to the development of ITP, contributing to platelet phagocytosis and B‐cell dependent auto‐antibody production.^13^ Circulating monocytes could reflect different inflammatory states.^14^, ^15^ It is known that expanded CD16^+^ monocytes may have a role in the pathological process of ITP.^16^ Recently, a theory of M1 and M2 monocytes, mirroring the M1 and M2 macrophages, has been proposed.^17^ Monocytes with double positive for Chemokine‐X3C‐receptor‐1 (CX3CR1) and CD163 have been precisely defined as M2 monocytes in rheumatoid arthritis, type 2 diabetes,^18^ hypercholesterolemia and atherosclerosis.^19^

Accumulating evidence indicated the participation of DNA methylation in the regulation of macrophage polarization.^20^ The initiation of peroxisome proliferator‐activated receptor‐γ (PPARγ), a crucial transcriptional factor for M2 macrophages,^21^ might partially attribute to the demethylation status of its promoter, which is featured by cytosine‐phosphate‐guanosine (CpG) islands.^22^, ^23^ The nucleotide binding oligomerization domain‐like receptor protein 3 (NLRP3) inflammasome was subsequently suppressed following PPARγ activation, and ultimately leading to M2 macrophages expanding with M1 macrophage suppression.^24^, ^25^ In present study, we demonstrated that DNA demethylation induced by low‐dose DAC could regulate M2 macrophage polarization in a PPARγ‐dependent way via suppressing NLRP3 inflammasome in ITP patients. In addition, ITP murine model was utilized to explore the effects and underlying regulation mechanisms of low‐dose DAC in vivo. These insights would shed new light on the pharmacological targets of DAC for the treatment of ITP.

## 2 MATERIAL AND METHODS

### 2.1 Patients and controls

A total of 36 newly diagnosed ITP patients (including 21 females and 15 males; age range 28−80 years, with the median age of 52 years) were enrolled in this study between June 2017 and May 2021 at the Department of Hematology, Jinshan Hospital and Zhongshan Hospital, Fudan University, China. All enrolled patients were diagnosed based on the recommended criteria of ITP timing for intervention (peripheral blood platelet count <30 × 10^9^/L, and exclude secondary ITP, pregnancy‐associated ITP, and thrombocytopenia related to other hematological disorders), and received HD‐DXM therapy (40 mg/day × 4 consecutive days).^1^, ^3^ By day 10 after treatment, blood samples were collected. Platelet counts ≥100 × 10^9^ /L was defined as complete response (CR), 30 × 10^9^ /L to 100 × 10^9^ /L or at least twice of the baseline was defined as partial response (PR), or else no response (NR). Twenty healthy controls (HC) (10 females and 10 males; age range 33−54 years, with a median age of 38 years) were recruited during the same study period, with a median platelet count of 234 × 10^9^ /L (range 173−305 × 10^9^ /L). The clinical characteristics of enrolled individuals were summarized in Table 1.

**TABLE 1:** Clinical characteristics of enrolled individuals.

Characteristics	ITP patients	Healthy Controls	*p‐*Value
**Cases**	36	20	
**Gender, number (%)**			.923
Famle	21 (58.3)	10 (50)	
Male	15 (41.7)	10 (50)	
**Age, years median (range)**	52 (28, 80)	38 (33,54)	.512
**Platelet counts (×10^9^/L), median (range)**	12.5 (3,29)	234 (173,305)	<.001
**Response to HD‐DXM regimen, number (%)**		**–**	
CR	26 (72.2)	**–**	
PR	2 (5.6)	**–**	
NR	8 (22.2)	**–**	

Abbreviations: CR, complete response; PR, partial response; NR, no response.

### 2.2 Passive ITP mice model and experimental grouping

Female, inbred C57BL/6 mice (*n* = 18, weighing 20 g, age 6−8 weeks) were purchased from Slac Laboratory (Shanghai, China). The monoclonal antibody anti‐mouse CD41 (clone MWReg30; BD Biosciences, USA) was injected intraperitoneally to induce passive ITP murine model.^26^, ^27^ These mice were randomized to 3 groups (*n* = 6 per group): control (injection of normal saline), ITP (injection of CD41 antibody), and DAC group (injection of CD41 antibody and DAC at .25 mg/kg/day, from day 1 to day 5). A total of 10 μl of blood was collected by saphenous vein bleeding daily from each animal for routine analysis of blood.

### 2.3 Isolation of CX3CR1^+^ monocytes

Peripheral blood mononuclear cells (PBMCs) were collected from ITP patients and HCs using Ficoll–Hypaque centrifugation within 2 h after blood withdrawal. Anti‐CX3CR1 microbeads kit (Miltenyi Biotec, Germany) was used to enrich CX3CR1^+^ monocytes from PBMCs per manufacture protocol with purity greater than 90 % (Figure S1A).

### 2.4 Lentiviral constructs and infection

After transfecting 293T cells with PPARγ shRNA plasmid and ad‐FLAG‐KLF4 plasmid (synthesized by Zorin Biobolgical technology, China), lentivirus was harvested and to infect THP‐1 cells (ATCC# TIB‐202). The puromycin‐selected infected THP‐1 cells were pooled for subsequent experiments after validating the effective knockdown or overexpression by RT‐qPCR and western blot (Figure S2A,B).

### 2.5 Cell culture and treatment

The femur and tibia of C57BL/6 mice were used to isolate the bone marrow cells, which were then cultured for seven days in RPMI‐1640 medium (Life Technologies, UK) with 10% fetal bovine serum (FBS) (Gibco, USA), 1% streptomycin and penicillin (Solarbio, China), and 30% L929 culture supernatant medium to differentiate them into bone marrow derived macrophages (BMDM).^28^ Fresh media supplemented with the polarizing cytokines were added to modulate M1 or M2 macrophage polarization for further 2 days (M1: 100 ng/mL LPS; M2: 20 ng/mL IL‐4 and IL‐13).

Recombinant human M‐CSF (10 ng/mL × 5 days) and phorbol 12‐myristate 13‐acetate (50 ng/ml, PMA, Sigma‐Aldrich) for 2 days were utilized to stimulate CX3CR1^+^ monocytes and THP‐1 cells separately into macrophages. After treatment with DAC (5 μM)^29^ or phosphate buffer saline (PBS) (used as solvent control) during the differentiation period, IL‐4 and IL‐13 were added to induce the differentiation of M2 macrophages for 3 days. The purity and viability were examined by flow cytometry (Figure S1B,C).

### 2.6 Immunophenotypic characterization by flow cytometry

M2 monocytes were defined as CX3CR1^+^ CD163^+^. The phenotype of M2 macrophages polarized by THP‐1 was analyzed by CD14^+^ CD163^+^. M1 macrophages were analyzed by F4/80^+^ CD86^+^ and F4/80^+^ CD206^+^ represented M2 macrophages in spleen. The FcγR were analyzed by CD64 (FcγRI), CD16 (FcγRIII) and CD32 (FcγRII). After co‐culture of the polarized M2 macrophages and CD4^+^ T cells, Treg cells were defined as CD25^+^ Foxp3^+^ and IL‐17^+^ represented Th17 cells. All antibodies were bought from BioLegend, USA. CD206, Foxp3, and IL‐17 were intracellularly stained by Foxp3 fixation/permeabilizing kit (BD PharMingen, USA). All samples were operated per the manufactures’ instructions. Expression analyses were performed using FACS Aria III flow cytometer (BD Biosciences, USA). All data were analyzed and imagined by FlowJo 10.0.7 software.

### 2.7 Western blots analysis

After lysing cells in RIPA lysis buffer (Beyotime, China) containing protease inhibitors (Thermo Fisher, USA), the protein extracts (20 μg) were performed for immunoblots. Primary antibodies used were: caspase‐1 and cleaved caspase‐1 (All from Santa Cruz, Biotechnology, USA), PPARγ, Arg‐1, DNMT3b, KLF4, NLRP3, GAPDH (All from Cell Signaling Technology, USA). After combined with HRP‐conjugated secondary antibodies (Cell Signaling Technology, USA), the protein expressions were detected by enhanced chemiluminescence (Beyotime, China).

### 2.8 Real‐time quantitative PCR

Total RNA was extracted from CX3CR1^+^ monocytes and THP‐1 cells using TRIzol Reagent (Invitrogen, USA), reverse transcribed into cDNA using PrimerScript RT Master Mix (Yeasen, China) and then processed for RT‐qPCR using SYBR Premix Ex Taq (Yeasen). The sequences of primers were shown in Table 2.

**TABLE 2:** Gene‐specific primers of qPCR and bisulfite‐sequencing PCR (BSP).

Primers	Forward primer (5′−3′)	Reverse primer (5′−3′)
*gapdh*	AACAGCCTCAAGATCATCAG	AGTCCTTCCACGATACCAA
*pparγ*	ACCAAAGTGCAATCAAAGTGGA	ATGAGGGAGTTGGAAGGCTCT
*pparγ* for BSP	AATTTTGGAGTAGGGTGTTTG	TTCCACCAAAAAACCTAAAATT
*arg‐1*	GTGGAAACTTGCATGGACAAC	AATCCTGGCACATCGGGAATC
*il‐10*	TCAAGGCGCATGTGAACTCC	GATGTCAAACTCACTCATGGCT
*dnmt3b*	CCCAGCTCTTACCTTACCATCG	GGTCCCCTATTCCAAACTCCT
*klf4*	CCCACATGAAGCGACTTCCC	CAGGTCCAGGAGATCGTTGAA

### 2.9 Cytokine IL‐10 analysis

Quantification of IL‐10 was performed using an ELISA kit (R&D Systems, USA) as per the manufacturer's instructions for plasma harvested from ITP patients, HCs and mice.

### 2.10 Proliferation and differentiation assay

CD4^+^ T cells from splenocytes were magnetically isolated by a Mouse CD4 T Cell Isolation Kit (BioLegend, USA), labelled with 5, 6 carboxyfluorescein diacetate succinimidyl ester (CFSE, 2.5 μM, Sigma‐Aldrich) and cocultured with bone marrow‐derived M2 macrophages. Recombinant anti‐mouse CD3 and CD28 (eBioscience, USA) were used to stimulate cells for 6 days for proliferation analysis and for 3 days for differentiation of CD4^+^ T cells. In addition, the pretreatment of cell stimulation cocktail (Invitrogen, CA) for 5 h was needed for T cell differentiation analysis.

### 2.11 Phagocytosis assay

Bone marrow‐derived M1 macrophages from varied group were cultured with Latex Beads‐Rabbit IgG‐DyLight 633 Complex at a 1:100 dilution for 2 h. Fluorescence was imaged on a confocal fluorescence microscope (Olympus, Japan) or detected by flow cytometry.

### 2.12 Immunofluorescent double staining

For immunofluorescent double staining of M1 (F4/80^+^ CD86^+^) and M2 (F4/80^+^ CD206^+^), paraffin‐embedded spleen sections were processed with deparaffinized, rehydrated and antigen retrieved. After fixing in 4 % paraformaldehyde for M1 (CD68^+^ CD86^+^) and M2 (CD68^+^ CD163^+^), THP‐1 cells were permeabilized with 1 % Triton X‐100, then blocked and incubated overnight with primary antibodies at 4°C, followed by staining with Alexa Fluor‐conjugated secondary antibody (1:500). The following primary antibodies were used: CD68, CD163, F4/80, CD86 (Cell Signaling Technology, USA) and CD206 (Abcam, USA). Cells were imaged with a Nikon Eclipse 800 upright epifluorescence microscope (Nikon Instruments, NY).

### 2.13 Detection of reactive oxygen species generation

MitoSOXTM reagent working solution (Invitrogen, CA) was used to incubate cells for 10 min at 37°C to detect reactive oxygen species (ROS) generation. Hoechst 33342 was subsequently added to identify the nucleus. The stained cells were observed using confocal fluorescence microscope.

### 2.14 DNA methylation analysis

Total DNA was isolated from CX3CR1^+^ monocytes using the DNA Mini Kit (Generay, China) for DNA methylation analysis of the ppr promoter. Using the EpiTect bisulfite Kit (Qiagen, Germany), complete bisulfite conversion of the DNA was then carried out. Subsequently, the bisulfite‐sequencing PCR (BSP) was used for testing the methylation status of pparγ CpG Island. After amplifying the 161‐bp region (−1625 to −1785) of CpG rich region in the upstream of the pparγ promoter transcription, the PCR products were subsequently purified and cloned for Sequencing (Life Technologies, USA) by using the pTG19‐T (Qeneray). To ensure the accuracy, five distinct clones from each sample were sequenced by an ABI 3730 XL DNA Analyzer. Then, using QUMA (available at http://quma.cdb.riken.jp/), the sequences were examined for methylated CpG regions. The primer sequences used for BSP of the pparγ promoter are provided in Table 2.

### 2.15 High‐throughput cut&tag

The enzyme‐tethering strategy known as CUT&Tag^30^ was performed to confirm that KLF4 binds to the promoter of pparγ based on the instructions for the Hyperactive Universal CUT&Tag Assay Kit (Vazyme, China) with minor optimizations. After harvesting, cocultured cells with activated ConA Beads for 10 min. Primary antibody (1:100) was added for incubating overnight at 4°C. Subsequently, cells were incubated with a corresponding secondary antibody (1:100) at RT for 60 min. After removing the supernatant by shot spin, Dig‐300 Buffer containing pA/G‐Tnp adapter complex was added and incubated for 1 h. Then incubated cells in 1× TTBL for fragmentation of tagmentation sample. After extracting DNA, N5 and N7 were added to perform library amplification. Illumina was finally used for high‐throughput sequencing.

### 2.16 Statistical analysis

The SPSS 19.0 programwas used to conduct all statistical analyses. The data were expressed as mean ± SD or median (range). Based on the normality results assessed by Shapiro‐Wilk W test, pairwise comparisons were analyzed by student's *t* test or Wilcoxon rank‐sum (Mann–Whitney) test. One way analysis of variance with the least significant difference test for post hoc multiple comparisons or Kruskal‐Wallis test were used for comparisons among multiple groups depending on whether the data had a normal distribution or not. For all tests, two‐tailed *p* values less than .05 were considered statistically significant.

## 3 RESULTS

### 3.1 M2 monocytes expanded after HD‐DXM treatment among the CR group

**FIGURE 1:**
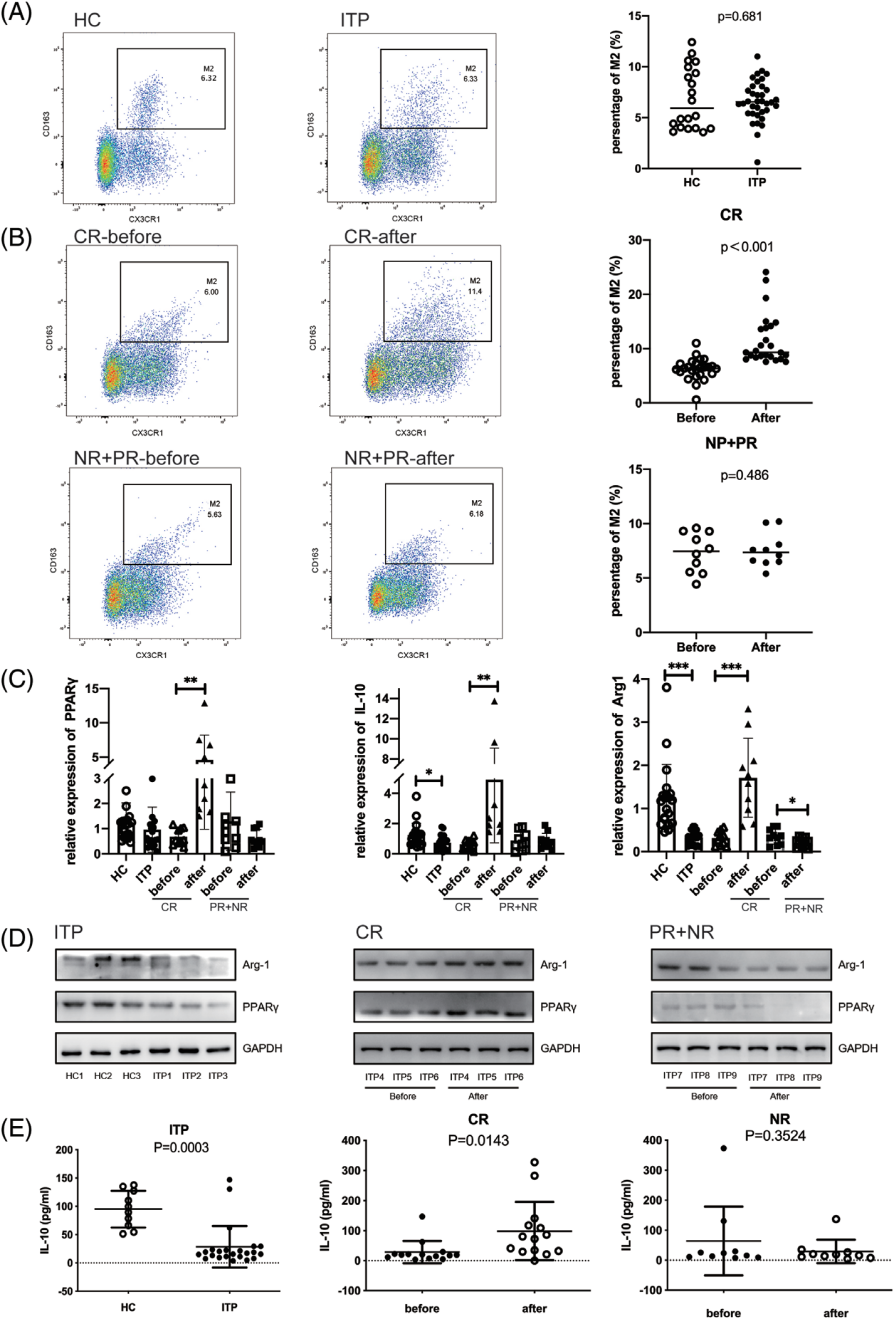
M2 monocytes expanded after HD‐DXM treatment in the CR group.(A) Representative dot plots of CX3CR1^+^ CD163^+^ M2 in the PBMCs of HC (*n* = 20) and ITP patients (*n* = 36).(B) Changes in the M2 monocytes among the CR group (*n* = 26) and PR+NR group (*n* = 10) monitored before and after treatment.(C) The mRNA expression level of PPARγ, IL‐10 and Arg‐1 in CX3CR1^+^ cells from HC, ITP patients, CR and PR+NR group (before and after treatment). (D) Representative immunoblots for PPARγ, Arg‐1 and GAPDH in CX3CR1^+^ cells from HC, ITP patients, CR and PR+NR group (before and after treatment).(E) Plasma cytokine IL‐10 is measured by Elisa. Data represent the mean ± SD. All experiments were repeated three times. ^∗∗^ *p* < .01; ^∗∗∗^ *p* < .001.

The percentages of M2 monocytes were analyzed in HCs and ITP patients. No significant difference was detected in M2 monocytes among all ITP patients compared with HC (6.617 ± 1.981 vs. 6.893 ± 3.013, *p* = .681, Figure 1A), while a significant elevation was verified after HD‐DXM treatment among the CR group (6.241 ± 1.970 vs. 11.580 ± 4.628, *p* < .001, Figure 1B). As CX3CR1^+^ monocytes harbored higher M2 specific markers (CD68 and CD163),^31^ they were further purified and then used to deeply probe into its function. The expression of both Arg‐1 and IL‐10 in CX3CR1^+^ monocytes, together with the cytokine IL‐10 secreted in the plasma were extremely down‐regulated in ITP patients. After HD‐DXM treatment, only patients among the CR group revised this phenomenon (Figure 1C–E, Figure S3A–C). The percentages of M1 monocytes were also detected in ITP patients and HCs, M1 monocytes were increased in ITP patients compared with HCs, and declined after treatment to achieve CR (Figure s4). In addition, the expression of PPARγ was disrupted in the ITP group. After HD‐DXM treatment, patients among the CR group retrieved the expression of PPARγ while not among the PR+NR group (Figure 1C‐D). These findings supported a significant expanded M2 monocytes among the CR group, and further hinted a correlation with PPARγ.

### 3.2 Low‐dose DAC promoted M2 macrophages polarization in a PPARγ‐dependent manner

**FIGURE 2:**
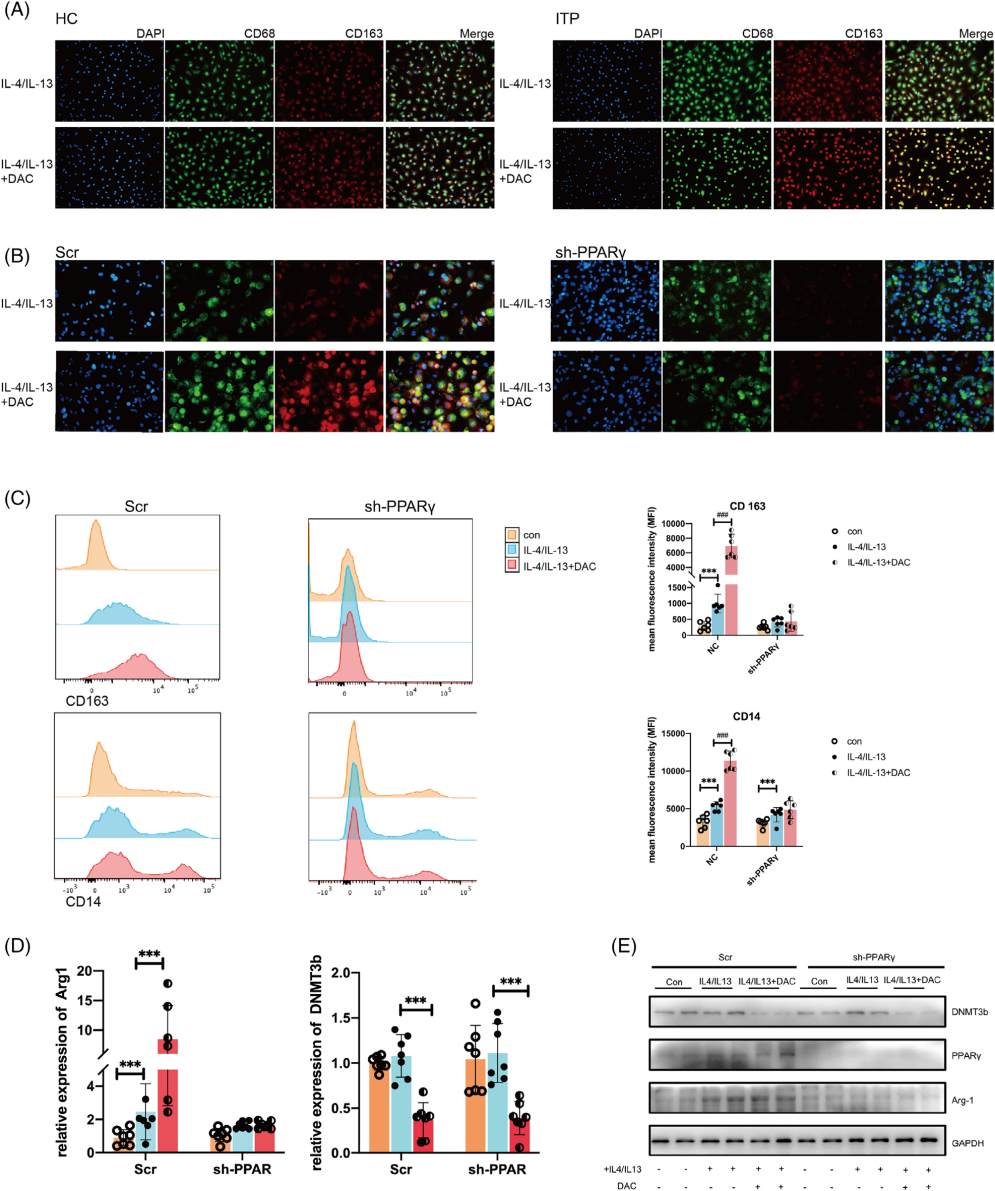
Low‐dose DAC promoted M2 macrophage polarization in a PPARγ‐dependent manner. (A) The CX3CR1^+^ monocytes derived from HC and ITP patients are stimulated with recombinant human M‐CSF (10 ng/mL) for 5 days and IL‐4/IL‐13 for 3 days to induce M2 macrophages. (B) Sh‐PPARγ and Scr THP‐1 cells are bulk differentiated to macrophages using 50 ng/ml PMA for 2 days. DAC (5 μM) or PBS (used as solvent control) was added during the differentiation period. Then add 20 ng/ml IL‐4/IL‐13 for further 3 days to induce M2 macrophages. The M2 macrophage polarization is determined by double positive CD68 (green) and CD163 (red). DAPI (blue) represents the nuclei. (C) The expression of CD14 and CD163 of sh‐PPARγ and Scr THP‐1 cells under low‐dose DAC treatment was measured by FCM. (D) The mRNA expression level of Arg‐1 and DNMT3b in sh‐PPARγ and Scr THP‐1 cells in low‐dose DAC treatment for 3 days. (E) Representative immunoblots for PPARγ, Arg‐1, DNMT3b and GAPDH in sh‐PPARγ and Scr THP‐1 cells under low‐dose DAC treatment for 3 days. Data represent the mean ± SD of three replicates. ^∗∗∗^ *p* < .001; compared to Con; ^###^ *p* < .001, compared to IL‐4/IL‐13 group.

To assess the potential ability of low‐dose DAC for M2 macrophage polarization, the CX3CR1^+^ monocytes from ITP patients and HCs were similarly stimulated with IL‐4 and IL‐13 under the pre‐treatment of PBS or low‐dose DAC in vitro. Immunofluorescence double staining demonstrated that CX3CR1^+^ monocytes from ITP patients were more easily polarized to M2 macrophages compared to those from HCs, and could be further expanded by low‐dose DAC treatment (Figure 2A).

To investigate the underlying molecular mechanism, THP‐1 cells were cultured and polarized in vitro. Low‐dose DAC‐treated THP‐1 cells also showed a greater increase in M2 macrophages polarization, as demonstrated by double positive CD163 and CD68 detected by immunofluorescence and higher expression of CD14 and CD163 detected by flow cytometry (FCM), accompanied with the significantly enhanced protein expression of Arg‐1 and PPARγ (Figure 2B‐E). To further explore the role of PPARγ in the regulation of M2 macrophage polarization, the PPARγ‐knockdown THP‐1 cell line was developed. Under either the routine M2‐polarized microenvironment or the additional DAC treatment, reduced CD163 and fixed Arg‐1 expression in sh‐PPARγ THP‐1 cells were detected, indicating that PPARγ might act as an indispensable element in M2 macrophages polarization (Figure 2B–E, Figure S3D). Taken together, low‐dose DAC could promote M2 macrophage polarization in a PPARγ‐dependent way.

### 3.3 Weak binding between KLF4 and PPARγ promoter could be reversed by low‐dose DAC

**FIGURE 3:**
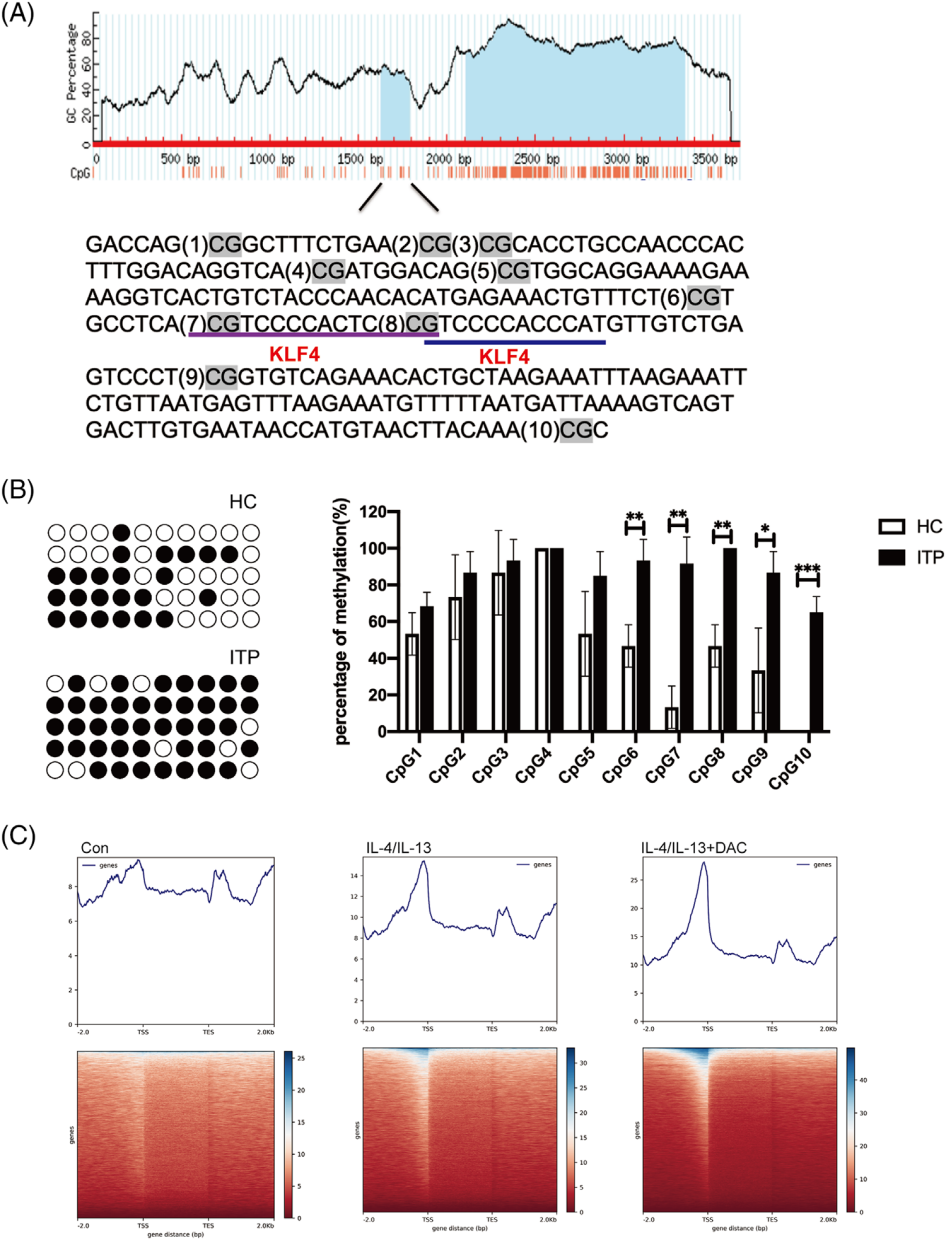
Higher methylation status of the KLF4 binding site of the PPARγ promoter could be reversed by DAC.(A) Bisulfite‐sequencing PCR (BSP) analysis of PPARγ methylation. The 161‐bp region (−1625 to −1785) containing 10 CpG residues in the CpG rich area upstream of transcriptional start site (TSS) was amplified and sequenced.(B) The representative plots of BSP analysis of DNA methylation (left) from HC and ITP patients. The percentage of methylated residues in the PPARγ promoter of CX3CR1^+^ cells of all HCs and ITP patients. (C) Combine trends manifested by Cut&Tag in THP‐1 cells under different treatments (control, IL‐4 + IL‐13, IL‐4 + IL‐13 combined with DAC). ^∗^ *p* < .05; ^∗∗^ *p* < .01; ^∗∗∗^ *p* < .001.

To explore the binding target of PPARγ, the methylation levels of CpG sites in the promoter of PPARγ were observed in CX3CR1^+^ monocytes from both ITP patients and HCs by BSP methods. Potential CpG islands of the PPARγ promoter were searched (Figure 3A). Consequently, 161 bp transcription initiation sites near the PPARγ promoter were chosen as the target sequence, which include 10 CpG sites. The whole methylation level of PPARγ was high among ITP patients compared with HCs (Figure 3B). Intriguingly, the PPARγ promoter was predicted to possess a functional KLF4 binding site at CpG7 and CpG8 by the Promo software and Harmonizome software. As a result, the methylation level of the KLF4‐binding site of the PPARγ promoter was significantly high in our data (Figure 3B, Figure s5). To determine the molecular basis for the supposed transcriptional synergy in vitro, the Cut&Tag assay verified that low‐dose DAC augmented the recruitment of KLF4 to the PPARγ promoter in THP‐1 cells (Figure 3C). Besides, low‐dose DAC showed no significant impact on the expression of KLF4 (Figure s6A,B). These results provided strong evidence supporting that the methylation level of the promoter of PPARγ was higher in CX3CR1^+^ monocytes among ITP patients. Once demethylated, the enhanced KLF4 binding in proximity to the promoter of PPARγ could promote M2 macrophage polarization.

### 3.4 Low‐dose DAC restored the balance of macrophages in ITP mice

**FIGURE 4:**
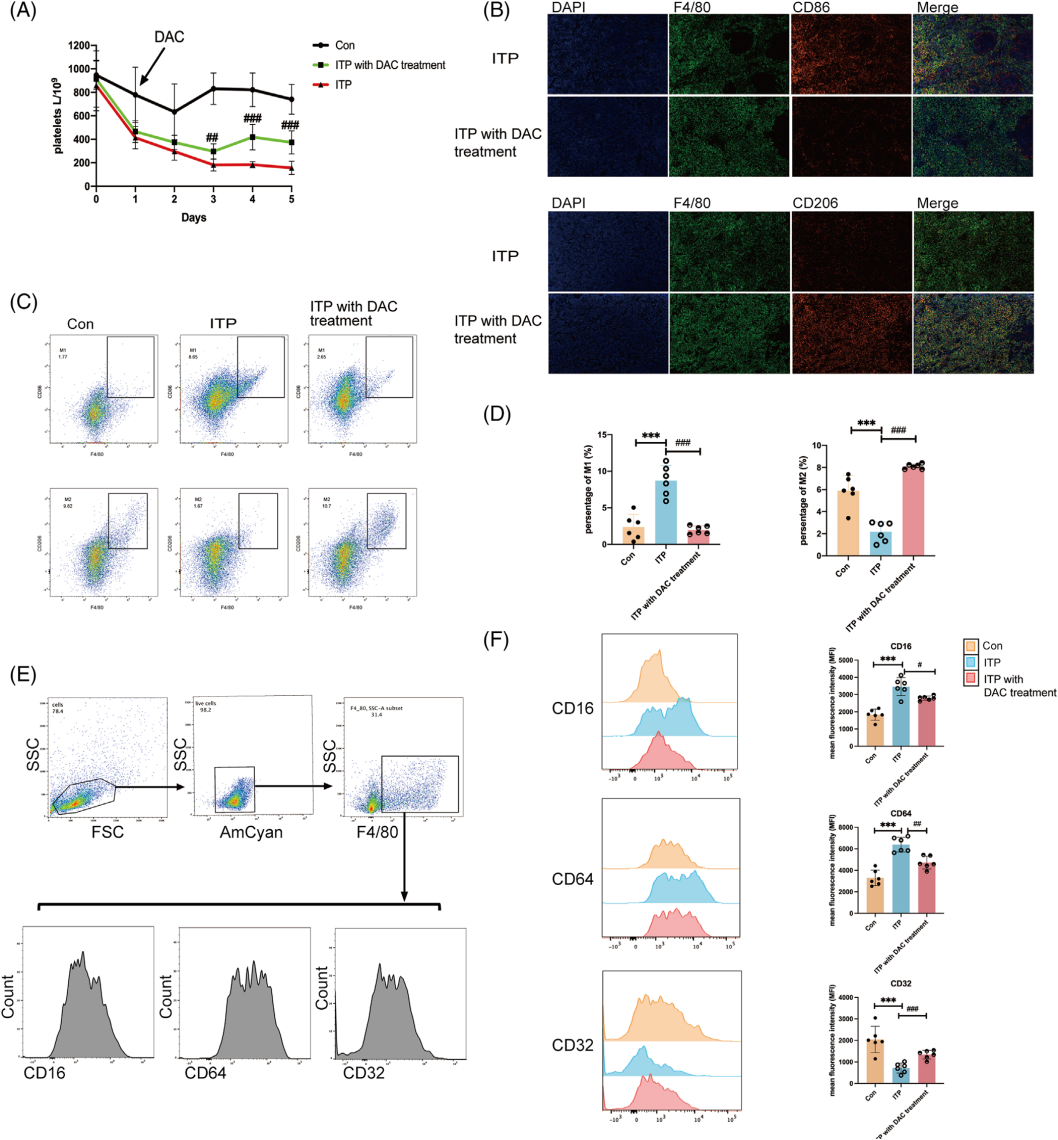
Low‐dose DAC restored the balance of macrophages in ITP mice. (A) Injecting C57B/6 mice with monoclonal antibody anti‐mouse CD41 intraperitoneally to achieve passive ITP mice model; low‐dose DAC treatment offset the decreased platelets in ITP mice on day 1. (B) On day 3, mice from different groups were executed and spleens were harvested for further analysis. Confocal fluorescence microscope at 400× magnification of macrophage (M1:F4/80^+^ CD86^+^, M2: F4/80^+^ CD206^+^) in spleen (blue, DAPI‐stained nuclei; green, F4/80‐stained; red, CD86‐stained, CD206‐stained). (C) Representative dot plots of M1 (F4/80^+^ CD86^+^) and M2 (F4/80^+^ CD206^+^) in the splenocyte of different groups (Control, ITP group, DAC treated group) and (D) the corresponding statistical graph. (E) The gating strategy of F4/80^+^ macrophages in spleen of mice among different groups (Control, ITP group, DAC treated group). (F) Mean fluorescence intensity of CD64 (FcγI), CD16 (FcγIII) and CD32 (FcγII) of F4/80^+^ macrophages in the splenocyte among different groups (Control, ITP group, DAC treated group) and the corresponding statistical graph. Data represent the mean ± SD. ^∗∗^ *p* < .01; ^∗∗∗^ *p* < .001, compared to control group; ^#^ *p* < .05; ^##^ *p* < .01; ^###^ *p* < .001, compared to ITP group.

To explore the effects of low‐dose DAC in vivo, the passive ITP mice which were caused by enhanced phagocytosis and proinflammatory of macrophages were established and exposed to DAC at .25 mg/kg/day. In ITP mice, the platelet counts had begun to decrease since day 0 injected with monoclonal antibody anti‐mouse CD41. Meanwhile, low‐dose DAC slowed the declining trend in platelet counts, especially at day 3 (296.7 ± 64.7 × 10^9^ vs. 181.7 ± 51.2 × 10^9^, *p* < .001, DAC vs. ITP, Figure 4A), indicating the therapeutical effect of low‐dose DAC for ITP.

Our data demonstrated that the treatment of low‐dose DAC decreased M1 macrophages (F4/80^+^ CD86^+^) and increased M2 macrophages (F4/80^+^ CD206^+^) in spleen when compared with ITP mice (Figure 4B), and so were results observed in FCM (Figure 4C,D, Figure s7A). Notably, the expression of activating receptor CD64 (FcγRI) and CD16 (FcγRIII) expanded while the inhibitory receptor CD32 (FcγRII) depressed in F4/80^+^ macrophage among ITP mice compared with the control. Furthermore, the expression of CD16 (FcγRIII) depressed while CD32 (FcγRII) expanded significantly when treated with low‐dose DAC (Figure 4E, F). These findings implied a strong correlation between the therapeutical effect of DAC and its role in regulating macrophage polarization.

### 3.5 The effect of low‐dose DAC on the immunomodulatory function of macrophages

**FIGURE 5:**
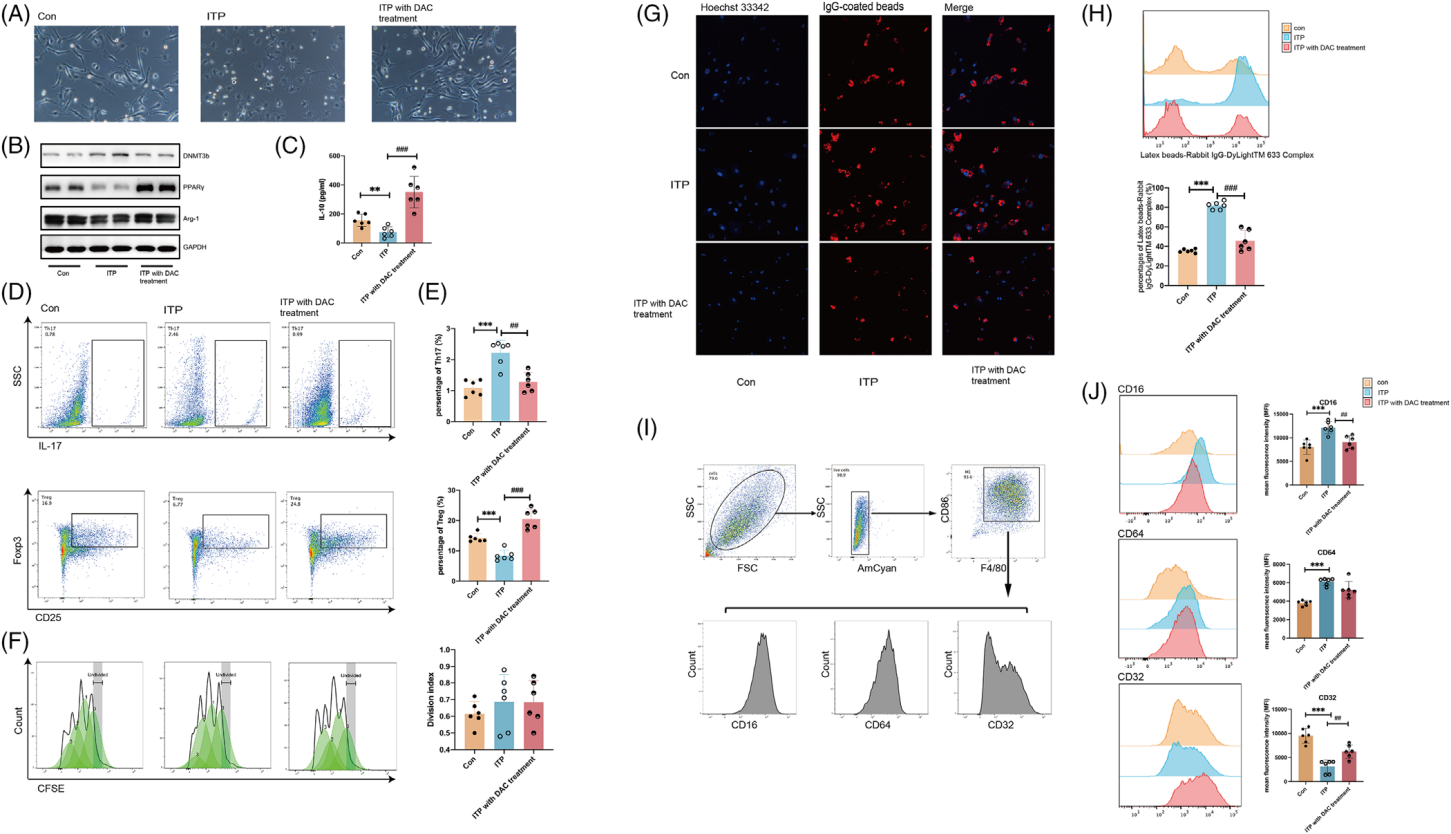
The effect of low‐dose DAC on the immunomodulatory function of macrophages. (A) BMDM derived from different groups under the same M2‐polarized microenvironment for 2 days. Phase contrast microscope is used to distinguish M1 and M2. (B) Representative immunoblots for PPARγ, Arg‐1, DNMT3b and GAPDH in bone marrow derived M2 from different groups (control, ITP group, DAC treated group). (C) Plasma cytokine IL‐10 is measured by Elisa. (D) Representative dot plots of Th17 (IL‐17^+^) and Treg (CD25^+^ Foxp3^+^) in the co‐culture system of CD4^+^ T cells with bone marrow derived M2 of different groups (control, ITP group, DAC treated group) and (E) the corresponding statistical graph.(F) CD4^+^ T cells were seeded in 96 well‐plate with bone marrow‐derived M2 of different groups. The cell division index is calculated based on the dilution of CFSE fluorescence measured by flow cytometry and represents the average number of cell divisions that CD4^+^ T cells in the original population have undergone. (G) Confocal fluorescence microscope at 400× magnification and (H) FCM verified the phagocytosis of bone marrow derived M1macrophages from different groups. (I) The gating strategy of bone marrow derived F4/80^+^ CD86^+^ M1 macrophages among different groups (control, ITP group, DAC treated group). (J) Mean fluorescence intensity of bone marrow derived M1 macrophages among different groups (Control, ITP group, DAC treated group) and the corresponding statistical graph. ^∗∗∗^ *p* < .001, compared to Control group; ^#^ *p* < .05; ^##^ *p* < .01; ^###^ *p* < .001, compared to ITP group.

Despite the increased M2 macrophage aggregation, whether the DAC treatment could modulate the immunomodulatory function of macrophages caught our attention. Polarized macrophages can be recognized by their shape and M2 macrophages exhibit an elongated shape compared with M1 macrophage.^32^ When compared with the ITP group, BMDM from DAC‐treated mice preferred M2 macrophage polarization under the same M2‐polarized microenvironment, with enhanced expression of both PPARγ and Arg‐1 (Figure 5A, B). In addition, the cytokine IL‐10 secreted in the plasma was significantly increased in DAC‐treated mice (Figure 5C). By detecting the immune regulation of M2 macrophages via testing the differentiation and proliferation of CD4^+^ T cells and by directly detecting the phagocytosis of M1 macrophages, we aimed to monitor the immunomodulatory function of macrophages. M2 macrophages derived from the DAC group promoted the Treg polarization and further reversed the Th17/Treg bias showed in ITP mice (Figure 5D, E, Figure s7B), although there was no effect on T cell proliferation (Figure 5F). As shown in Figure 5G, after low‐dose decitabine treatment, the phagocytosis of M1 macrophages significantly decreased compared with the increased phagocytosis in ITP mice. Meanwhile, CD32 (FcγRII) in M1 macrophages increased under low‐dose DAC treatment (Figure 5I, J). Together, low‐dose DAC has been proved to monitor the immunomodulatory function of macrophages.

### 3.6 The association of PPARγ against NLRP3 inflammasome with low‐dose DAC modulation

**FIGURE 6:**
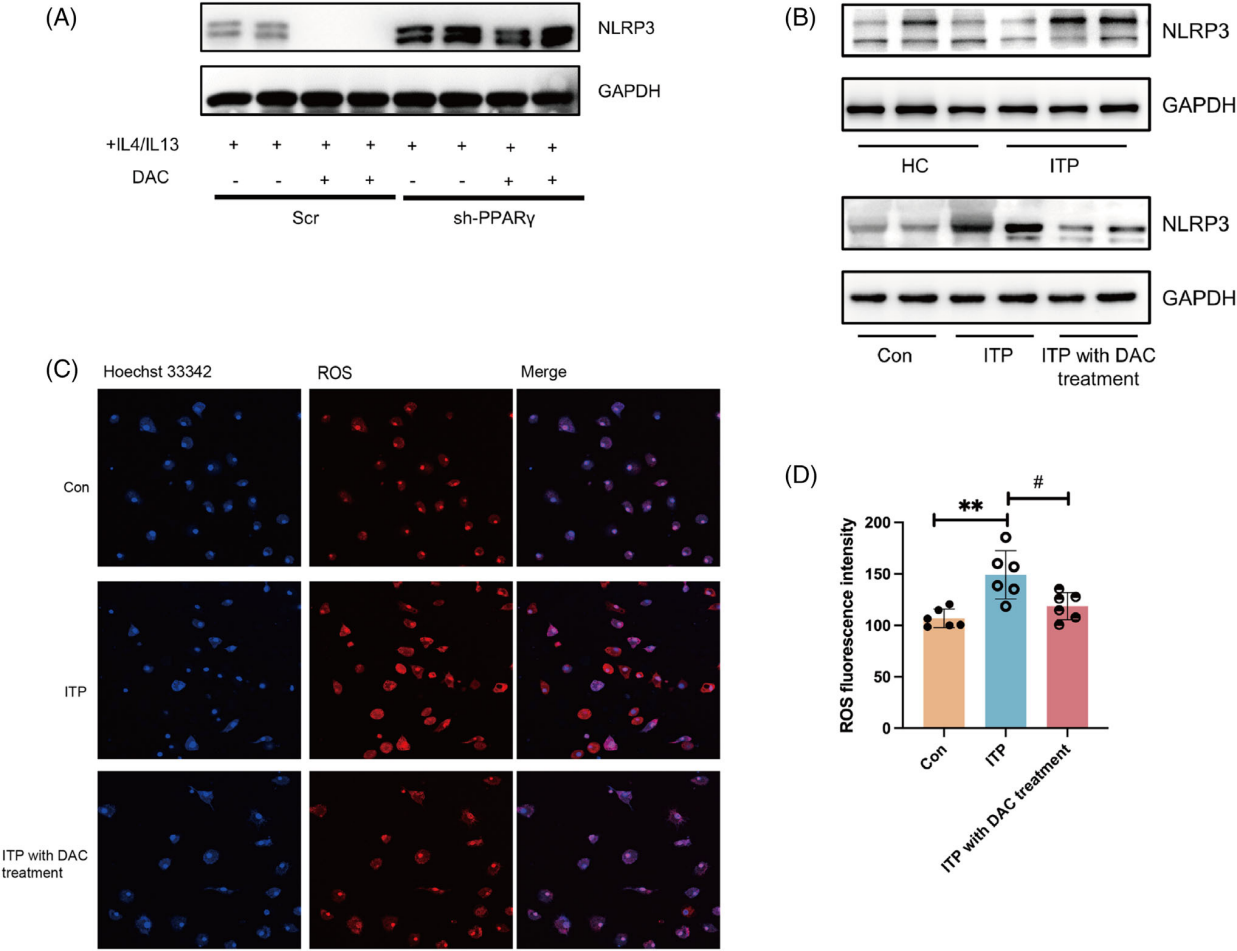
The association of PPARγ ameliorated NLRP3 inflammasome with low‐dose DAC modulation. (A) Representative immunoblots for NLRP3 in sh‐PPARγ and Scr THP‐1 cells under the stimulation of IL4 and IL13 for two days, with or without the treatment of low‐dose DAC. (B) Representative immunoblots for NLRP3 and GAPDH in CX3CR1^+^ cells of HC and ITP patients (upper) and in BMDM of different groups (lower) (control, ITP group, DAC treated group). (C) In BMDM of different groups, cellular mitochondrial ROS are stained with MitoSOX probe. (D) The quantification of ROS production. ^∗∗^ *p* < .01, compared to WT; #p < .05; compared to ITP group.

While the anti‐inflammatory role of PPARγ in macrophages has been revealed in attenuating NLRP3 inflammasome activation, which is crucial for M1 macrophage polarization, the precise events that occur downstream of PPARγ in M2 macrophage polarization remain obscure.^24^, ^33^ In the same way, we observed the knockdown of PPARγ increased expression of NLRP3 and NLRP3‐dependent caspase‐1 activation in THP‐1 cells when stimulated with LPS (Figure s8). Under the stimulation of IL4 and IL13, the knockdown of PPARγ also increased the expression of NLRP3 in THP‐1 cells, and low‐dose DAC decreased the NLRP3 only in the presence of PPARγ(Figure 6A). In CX3CR1^+^ monocytes among ITP patients, the protein expression level of NLRP3 was up‐regulated, which was further suppressed in BMDM after the DAC treatment in ITP mice model (Figure 6B). It is widely recognized that the activation of the NLRP3 inflammasome can be triggered by danger signals, such as ROS.^34^ As the classical activator for NLRP3, the levels of ROS in LPS‐stimulated BMDM cells from varied groups were detected. As described in Figure 6C, the BMDM group from ITP mice showed more ROS production, while DAC blunted the increase in the production of ROS. Combined, these results inspired us that PPARγ physiologically antagonizes NLRP3 inflammasome, which might be the underlying mechanism for the polarization of M2 macrophages induced by low‐dose DAC.

## 4 DISCUSSION

As an autoimmune hemorrhagic disease, ITP is portrayed by lacking platelet counts and increased bleeding risk. The heterogenous clinical symptoms and the incompletely understood pathophysiology make the therapeutic management of ITP challenging.^35^, ^36^ Our data suggested that the demethylation of PPARγ promotor could promote M2 macrophage polarization via enhancing KLF4 binding affinity and subsequent inhibition of NLRP3 inflammasome. Significantly higher DNA methylation level in PPARγ promoter especially the KLF4‐binding site was verified in M2 monocytes among ITP patients compared with HCs. Cut&Tag assay further unraveled low‐dose DAC could enhance KLF4 binding to the PPARγ promoter by demethylation, elucidating how low‐dose DAC induced M2 macrophage polarization in a PPARγ‐dependent way. Low‐dose DAC not only regulates macrophage polarization but also regulates the immunomodulatory function of macrophages, manifesting a positive therapeutical effect in ITP mice. Moreover, the suppressed expression of NLRP3 inflammasome induced by PPARγ might account for M2 macrophage polarization. These results suggested that regulation of M2 macrophage polarization, demethylation of PPARγ promoter, and suppressed NLRP3 inflammasome might be involved in the immunoregulatory effect of low‐dose DAC in ITP.

Macrophages are indispensable immune cells with great plasticity, presenting in two distinct states of activation: the pro‐inflammatory M1 macrophages and M2 macrophages with immunosuppressive functions.^37^ Not only the prevalence of M1 macrophage polarization, but CD16^+^ monocytes, which have been proved to expand and depress Treg cells, have also been involved in the pathogenesis of ITP.^38^ Recently, M2 monocytes have already been precisely defined in rheumatoid arthritis, type 2 diabetes^18^ and atherosclerosis.^19^ In the present study, we explored the M2 monocytes in ITP. Considering the lower expression level of Arg‐1 and IL‐10 among the ITP group, the M2 monocytes might be functionally invalid. After HD‐DXM treatment, only patients in the CR group showed the significantly increased M2 monocytes and elevated Arg‐1 and IL‐10, while the PR+NR group manifested the opposite. The vitro experiment further showed M2 monocytes from ITP patients possessed more potent chemotactic activity for M2 macrophage polarization. These data strengthened our hypothesis that M2 monocytes might act as a predictor for evaluating therapeutic effect.

As a regular demethylation regimen for myelodysplastic syndrome, DAC has been found to be advantageous in multiple myeloproliferative diseases such as Hodgkin lymphoma and acute myelogenous leukemia by regulating immune response.^39^, ^40^ Recently, growing evidence has indicated that low‐dose DAC‐induced M2 macrophage polarization could ameliorate acute lune injury^41^ and atherosclerosis,^42^ implying the potential role of DAC on immunological disorders. The aberrant DNA methylation has been found to participate in the pathophysiology of ITP,^43^ targeting the DNA methylation could be used as a new treatment for ITP. Since 2019, low‐dose DAC has been explored to treat adult patients with refractory ITP.^7^ By decreasing programmed cell death protein 1 on CD8^+^ T cells and restoring Treg function via inhibiting STAT3 or via induction of forkhead box P3 (Foxp3), low‐dose DAC could enhance the immunosuppressive function of Th and Tc cells in ITP.^44^, ^45^ Hou et al.^9^ also proved that low‐dose DAC promoted the megakaryocyte maturation via decreasing methylation status of tumour necrosis factor‐related apoptosis‐inducing ligand (TRAIL) promoter. To obtain unpolarized monocytes and induce them into M2 macrophages in vitro, BMDM, not splenic macrophages of mice were used for the experiments. Results confirmed that low‐dose DAC enhanced M2 macrophage polarization in both HC and ITP patients, especially in ITP. Considering the vital role of macrophages in the pathogenesis of ITP, the immunomodulation on macrophages further explained the therapeutic effects of DAC in ITP patients and provided more theoretical basis for the combination of DAC with other therapeutic drugs for the treatment of ITP.

These effects of low‐dose DAC on M2 monocytes might attribute to the higher methylation level of the PPARγ promoter of M2 monocytes in ITP patients. Intriguingly, the highly hypermethylated CpG7 and CpG8 were found to form the binding sites of KLF4, which has been previously proved to cooperate with PPARγ and to be critical for M2 macrophage polarization.^46^ Previous studies have suggested that KLF4 is a downstream protein of PPARγ,^47^, ^48^ further, the expression of KLF4 could also augment PPARγ expression.^49^ While in the present study, low‐dose DAC did not enhance the expression of KLF4. By utilizing the Cut&Tag assays, we found that low‐dose DAC successfully enhanced the KLF4 binding affinity with the PPARγ promoter, providing insight into dissecting its role in M2 macrophage polarization. Further, we suggest the downstream affections of PPARγwere mediated by NLRP3. NLRP3 has been revealed as the key factor of M1 macrophage polarization, and its activation has been further proved to be dependent on PPARγ activation and production.^50^ Similarly, we found that in M2 macrophages, low‐dose DAC decreased the NLRP3 depending on the presence of PPARγ, suggesting that PPARγ is the upstream regulating signal of NLRP3. However, the specific ways in how PPARγ acts on NLRP3 still need to be explored.

In addition, the depressed phagocytic function of M1 macrophage under the treatment of low‐dose DAC was also verified. Previously, Zambuzi et al.^51^ indicated that when exposed to Mycobacterium tuberculosis in vitro, DAC increased bacterial phagocytosis of monocytes and further hypothesized that these effects might attribute to the increased expression of IL‐8. Different from bacterial infection, a relative low expression of IL‐8 was observed among ITP patients.^52^ Together with the classic antibody FcγR‐dependent platelet phagocytosis,^53^, ^54^ the impaired phagocytic function of M1 macrophages might be explained by the increased expression of FcγRII.

To date, this is the first study to unfold the indispensable role of PPARγ in M2 macrophage polarization in adult ITP patients. Demethylation of the PPARγ promoter by low‐dose DAC was verified to enhance the KLF4 binding affinity, thereby shedding light on the mechanism of M2 macrophage polarization in ITP patients. The study revealed the underlying mechanism of M2 macrophage polarization of low‐dose DAC in ITP, which would provide novel insights for the treatment of ITP patients who are often suffered with disease relapse or refractory situations. However, findings of the study should be viewed with its limitations acknowledged. The study would be enriched if data from ITP patients before and after treated with DAC were available. Furthermore, although passive ITP mice model is believed to be powerful enough to study the role of macrophages played in the pathogenesis of ITP, active ITP mice model would be more convictive. As such, further studies are warranted.

## 5 CONCLUSION

Impaired polarization of monocytes/macrophages is involved in the immune pathogenesis of ITP. Low‐dose decitabine improved the polarization of macrophages in vitro and in vivo, and low‐dose DAC promoted M2 macrophage polarization due to the demethylation within the promoter of PPARγ thus enhancing KLF4 binding affinity in ITP. The study uncovered that DAC could act as an immunotherapeutic approach to restore the polarization of macrophages for relapse or refractory ITP and has the potential to achieve a sustained response in these patients. The study also provided evidence and mechanistic insights for DAC in the treatment of other autoimmune diseases.

## CONFLICT OF INTEREST STATEMENT

The authors declare no conflict of interest.

## Supporting information

Supporting Information

## Data Availability

All data used or analyzed during the current study are available from this published article and the corresponding supplementary information files.
