# Supplementary material for: Low‐dose decitabine promotes M2 macrophage polarization in patients with primary immune thrombocytopenia via enhancing KLF4 binding to PPARγ promoter
Source: Clin Transl Med. 2023 Jul 24;13(7):e1344. doi: 10.1002/ctm2.1344 (PMC10366349; doi:10.1002/ctm2.1344)

**Supplementary Figure 1. The purity and gating strategy of M2 macrophages.**

(A) The percentages of CX3CR1^+^ cells in isolated cells by anti-CX3CR1 microbeads kit from peripheral blood mononuclear cells (PBMCs). (B) The percentages of CD14^+^CX3CR1^+^ cells in Thp1 cells which were induced by IL4 and IL13 for two days. (C) The percentages of live cells in Thp1 cells which were induced by IL4 and IL13 for two days. (D) The gating strategy of M2 macrophages in PBMCs from immune thrombocytopenia (ITP) patients or healthy controls (HCs).

**Supplementary Figure 2. Effective knockdown PPARγ and overexpression KLF4 in THP-1 cells.**

The specific silencing PPARγ (A) and KLF4 overexpression (B) were evaluated by western blot and RT-qPCR.

**Supplementary Figure 3. Quantification of immunoblots.**

(A-C) Quantification of PPARγ and Arg-1 of immunoblots in CX3CR1^+^ cells from PBMCs among HCs and ITP patients (A), CR (B) and PR+NR (C) group (before and after treatment). (D) Quantification of DNMT3b, Arg-1 and PPARγ of representative immunoblots in sh-PPARγ and Scr THP-1 cells under low-dose DAC treatment. ^*^*p* < 0.05, ^**^*p* < 0.01, ^***^*p* < 0.001, ^#^*p* < 0.05.

**Supplementary Figure 4. The changes of M1 macrophages in HCs and ITP patients.**

M1 macrophages were defined as CD14^+^CD86^+^ cells. Representative dot plots of CD14^+^CD86^+^ M1 cells (left). Statistical plots showed the percentages of CD14^+^CD86^+^ M1 cells in the PBMCs of HC (n = 20) and ITP patients (n = 36), the CR group (n = 26) and PR+NR group (n = 10) before and after treatment. ^***^*p* < 0.001.

**Supplementary Figure 5. Representative plots of BSP analysis.**

Representative plots of BSP analysis of DNA methylation in the PPARγ promoter of CX3CR1^+^ cells from three HCs and three ITP patients.

**Supplementary Figure 6. The expression of KLF4 under the treatment of low-dose DAC in THP-1 cells.**

Western blot (A) and RT-qPCR (B) were used to evaluate the expression of KLF4 under varied treatment (none, IL-4 + IL-13, IL-4 + IL-13 + low dose DAC) in THP-1 cells.

**Supplementary Figure 7. The gating strategy of macrophages in mice and T cells.**

(A) The gating strategy of F4/80^+^CD86^+^ M1 macrophages and F4/80^+^CD206^+^ M2 macrophages in the spleens of ITP mice. (B) The gating strategy of IL17^+^ Th17 cells and CD25^+^Foxp3^+^ regulatory T cells in CD4^+^ T cells.

**Supplementary Figure 8. Western blot of NLRP3 and caspase-1 GAPDH in Thp1 cells.**

Representative immunoblots for NLRP3, caspase-1, cleaved caspase-1 and GAPDH in sh-PPARγ and Scr THP-1 cells which were stimulated with LPS for two days.

**Supplementary Figure 1.**


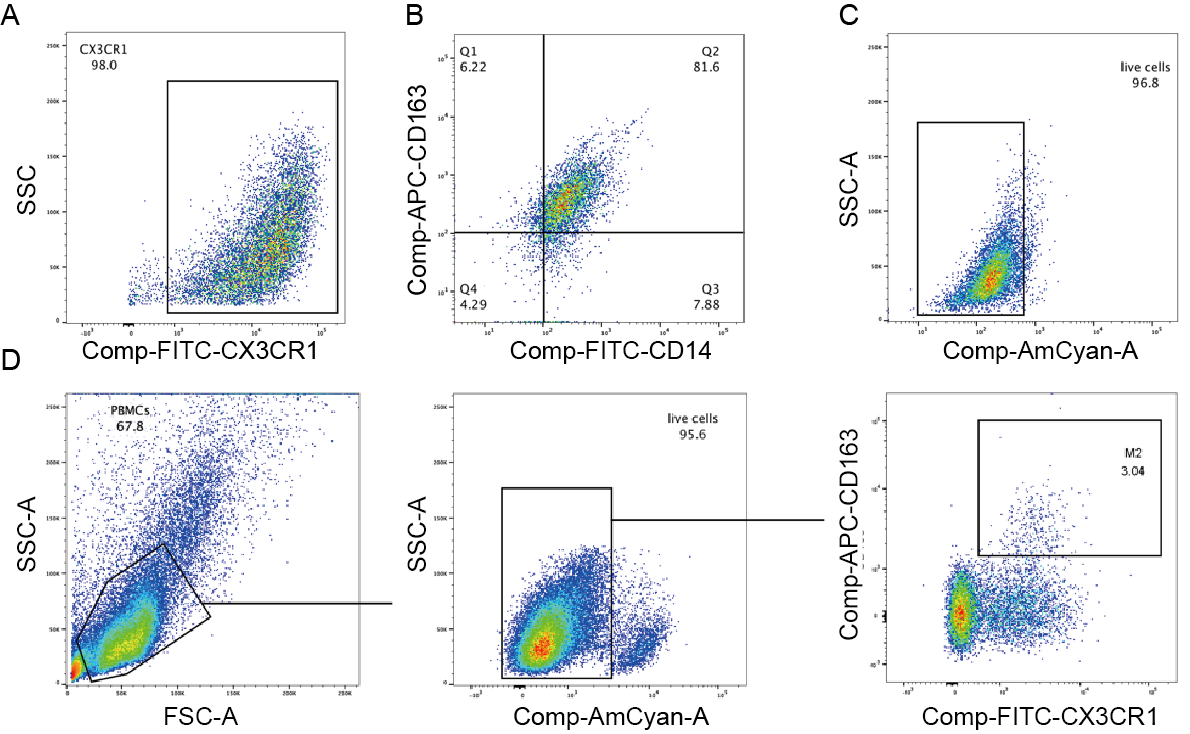


**Supplementary Figure 2**


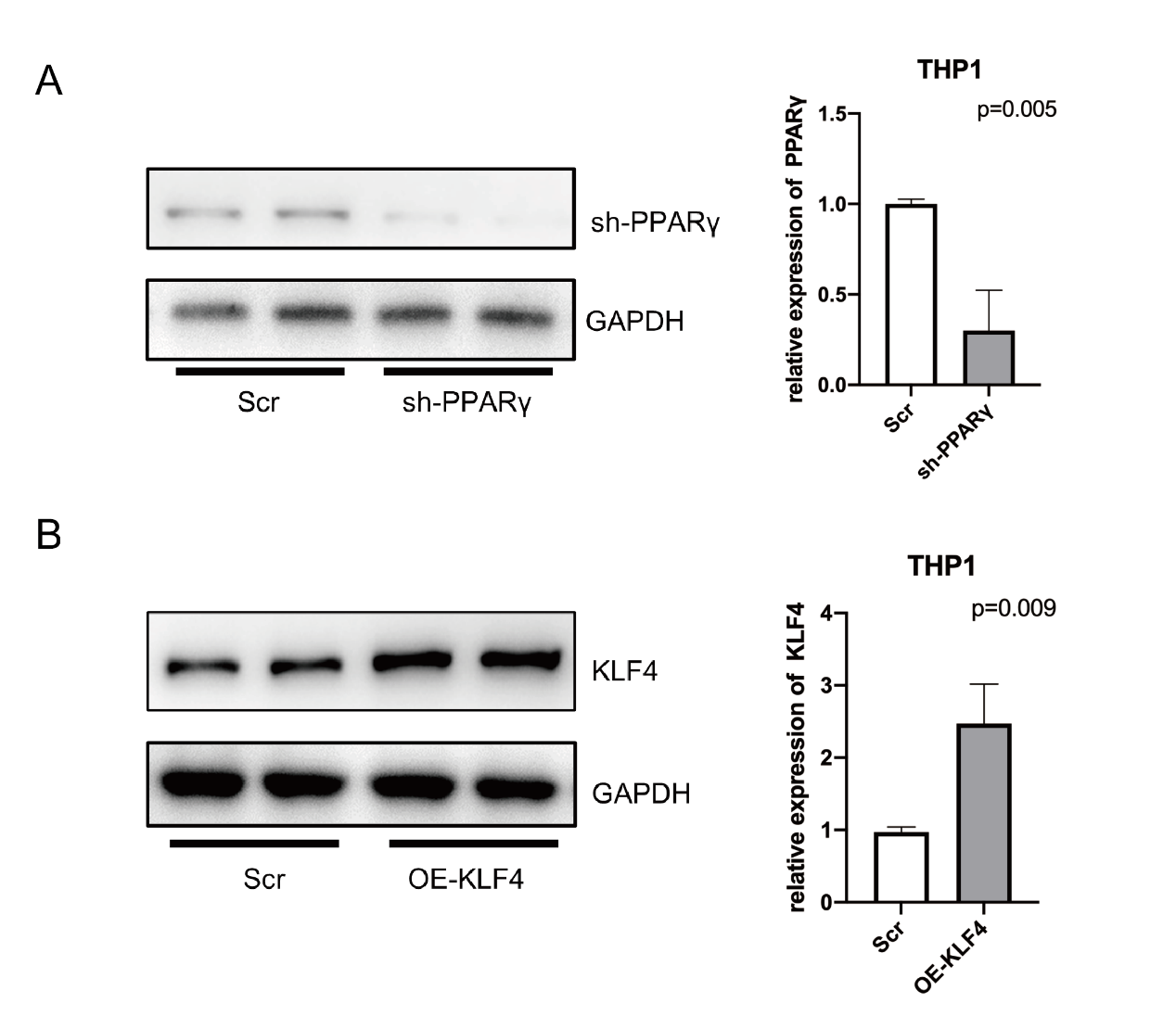


**Supplementary Figure 3**


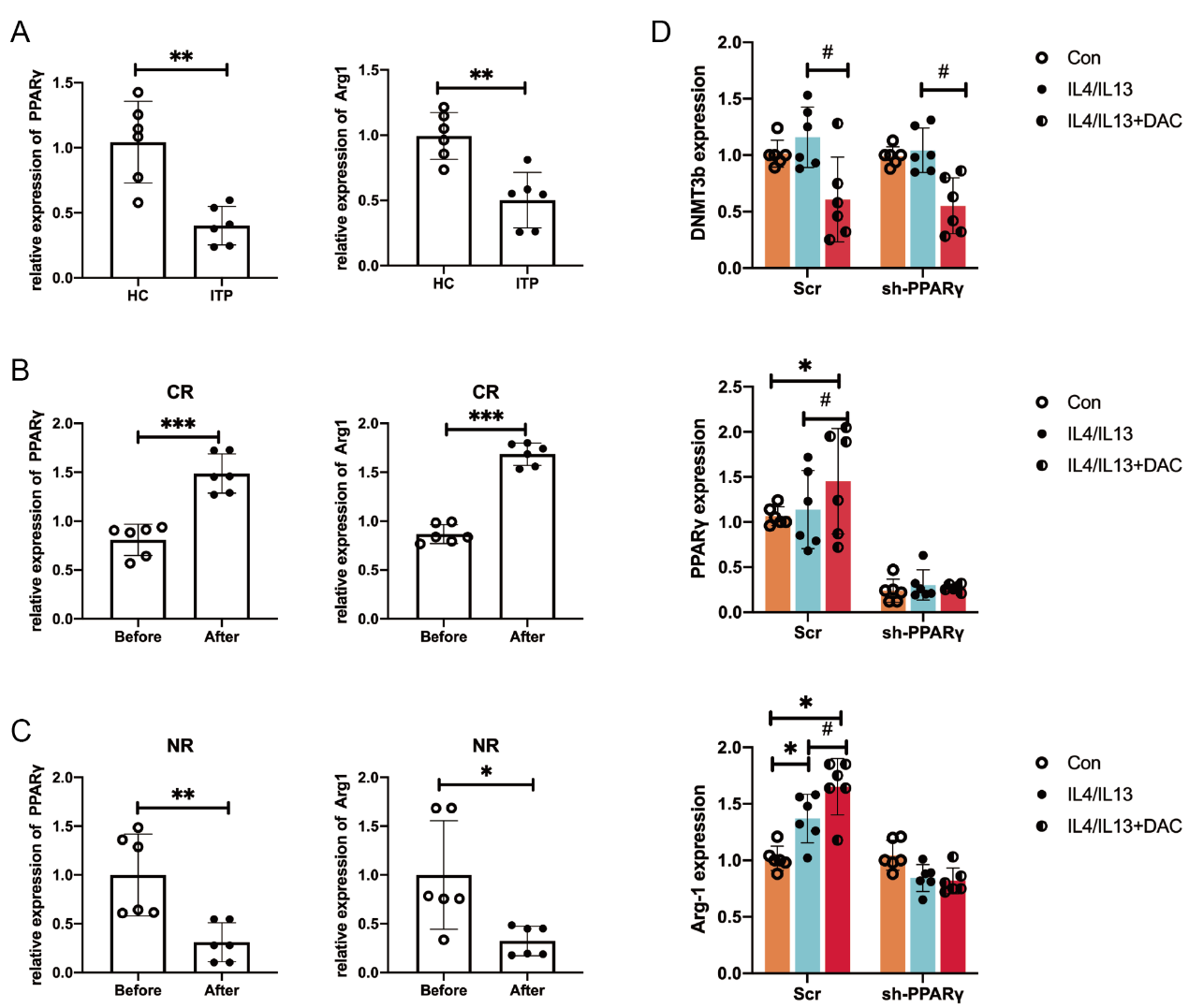


**Supplementary Figure 4**


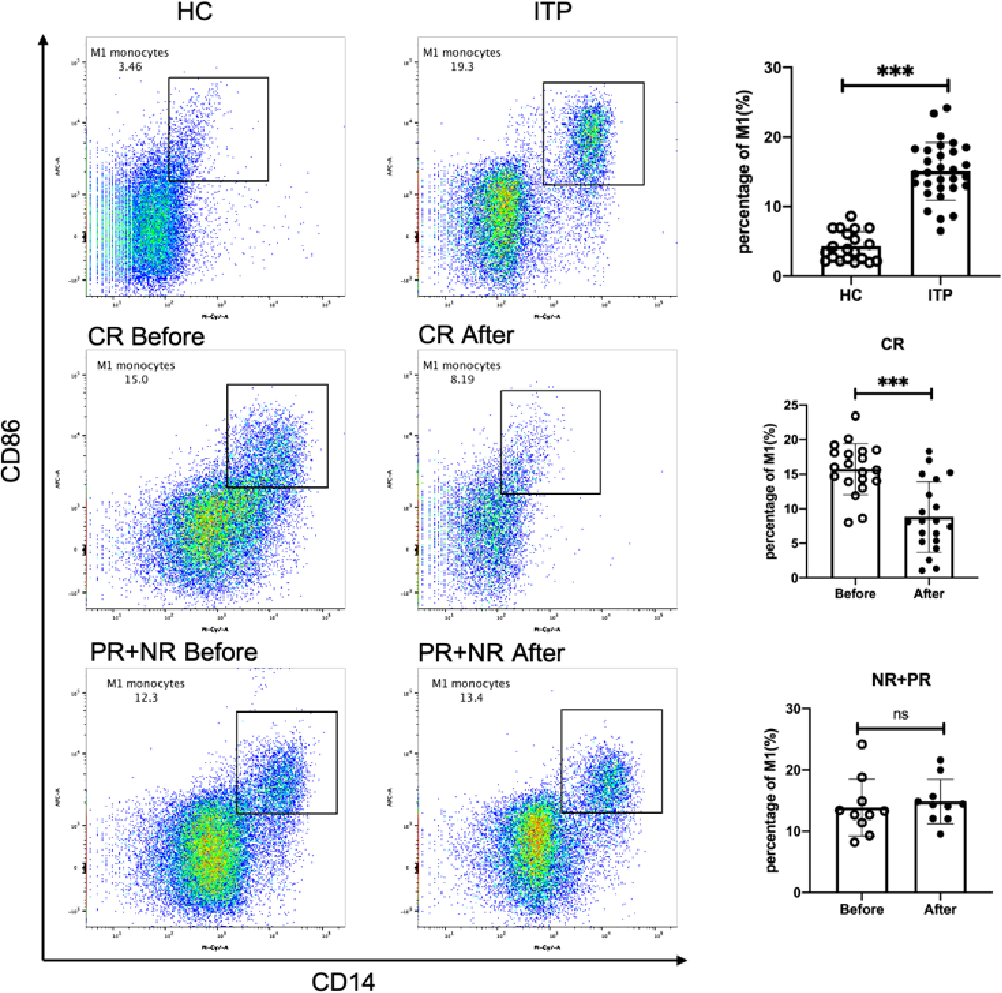


**Supplementary Figure 5**


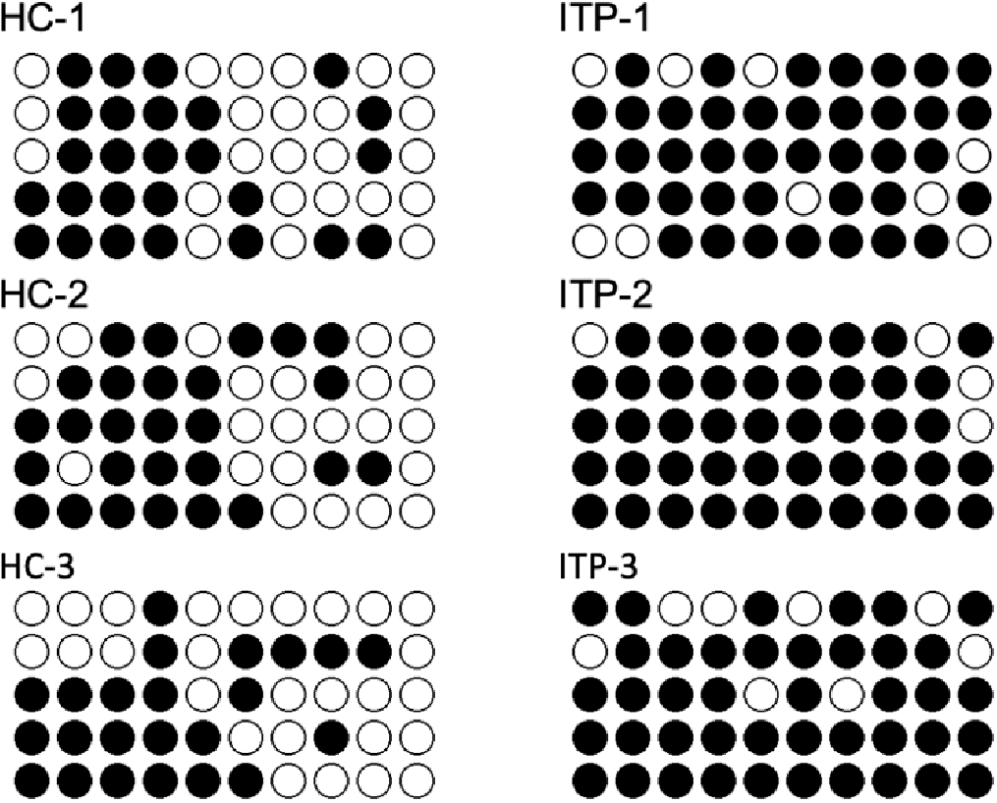


**Supplementary Figure 6**


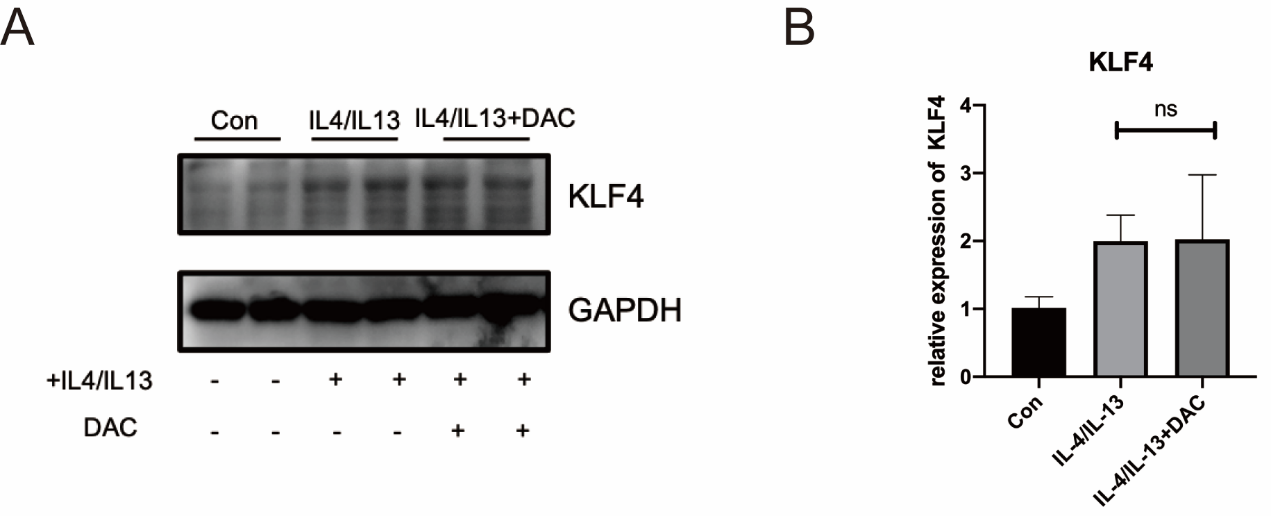


**Supplementary Figure 7**


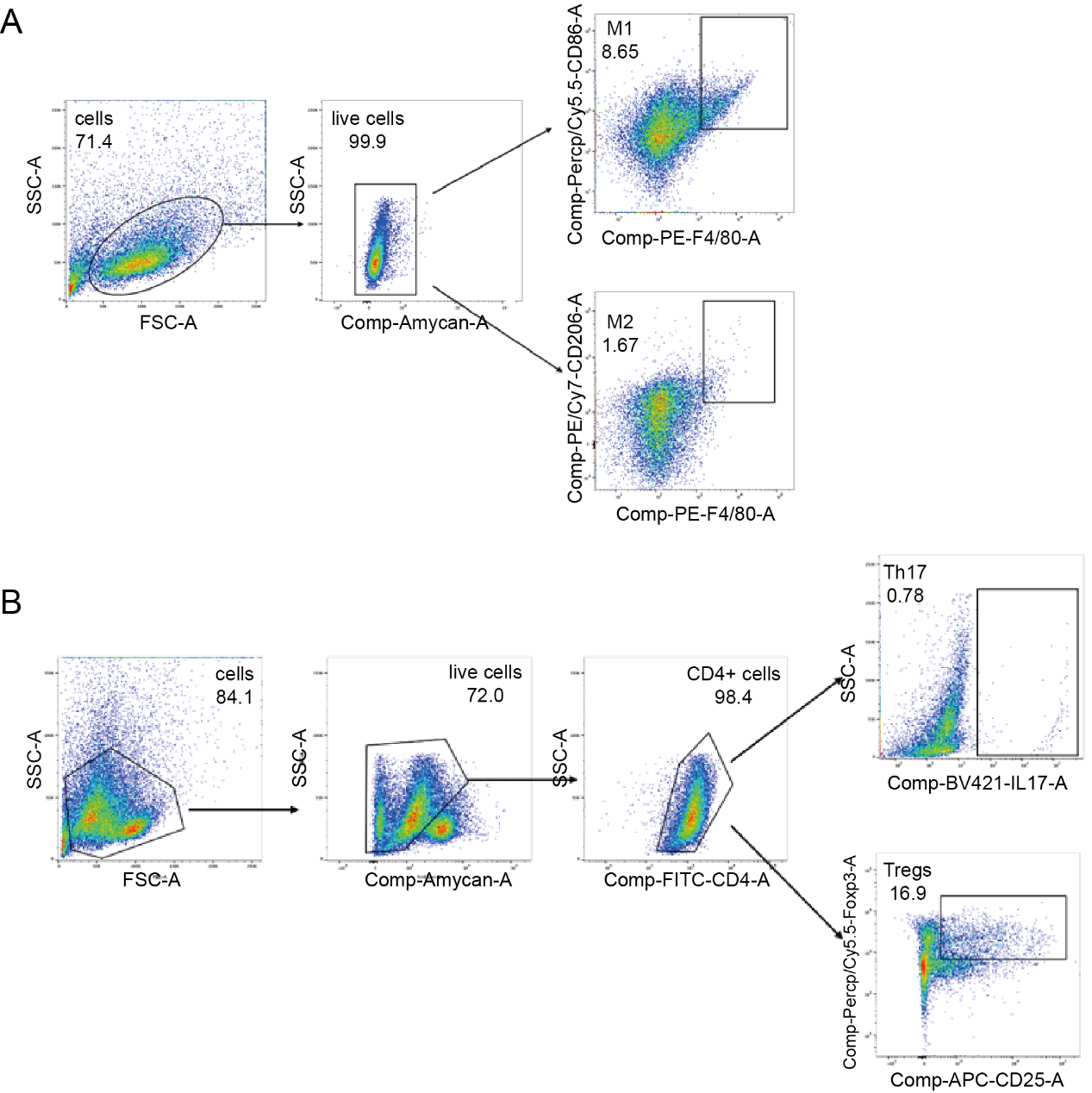


**Supplementary Figure 8**


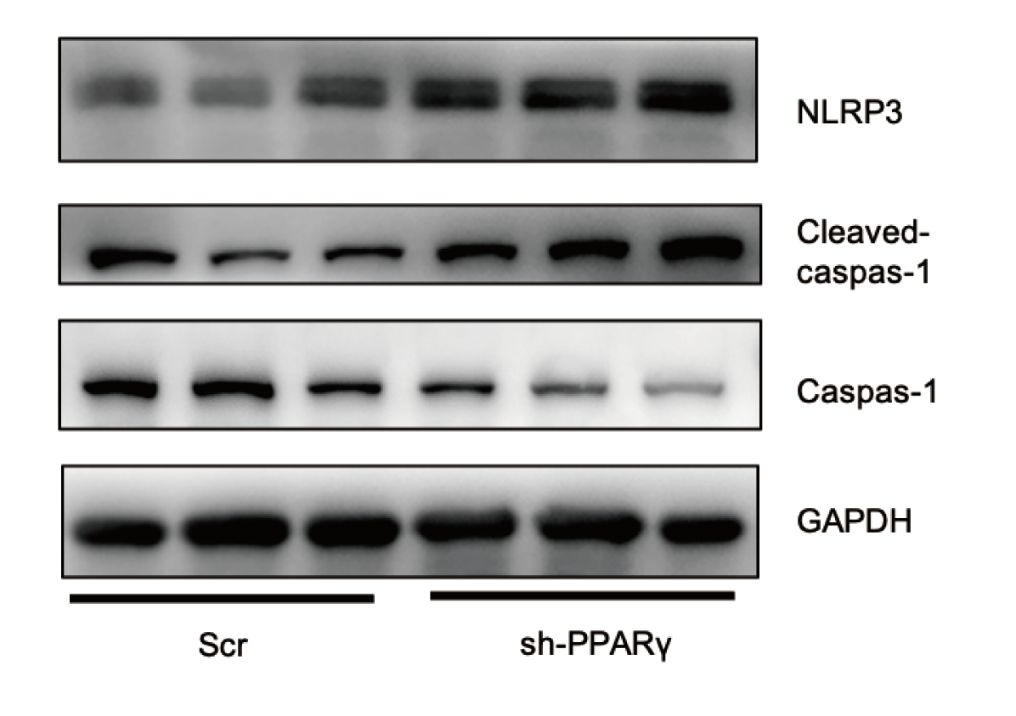

Supplement: Supplementary file 1 — Supporting Information [file CTM2-13-e1344-s001.docx]
